# Supplementary material for: Comparison of imaging-based single-cell resolution spatial transcriptomics profiling platforms using formalin-fixed, paraffin-embedded tumor samples
Source: Res Sq. 2025 Jan 17:rs.3.rs-5656204. Preprint. [Version 1] doi: 10.21203/rs.3.rs-5656204/v1 (PMC11774462; doi:10.21203/rs.3.rs-5656204/v1)
Supplement: Supplement 1 [file NIHPPRS5656204v1-supplement-1.pdf]

Supplementary Files

This is a list of supplementary files associated with this preprint. Click to download.

- SupplementaryTable1.xlsx
- SupplementaryTable2.xlsx
- SupplementaryTable3.xlsx
- SupplementaryFig1.pdf
- SupplementaryFig2.pdf
- Supplementary.Fig3.pdf
